# Clinical study on flipped classroom and mind map in newly recruited nurses’ pre-job training

**DOI:** 10.1186/s12912-022-00843-z

**Published:** 2022-03-29

**Authors:** Yingmin Liu, Yuyuan Li, Xueran Cui, Haikun Zhou, Jingjing Wang, Yan Zhang

**Affiliations:** Department of Nursing, The No.2 Hospital of Baoding, No. 338 Dongfeng West Road, Jingxiu District, Baoding, 071051 Hebei Province China

**Keywords:** Flipped classroom, Mind map, Pre-job training, Autonomous learning ability, Nurses

## Abstract

**Background:**

Traditional pre-job training mainly provides theoretical lectures and operational skill training for new nurses. However, it has a single teaching method, lacks in comprehensiveness and flexibility, and has unsatisfactory teaching effects. The purpose of this article is to evaluate the influence of the flipped classroom and mind map in the pre-job training of newly recruited nurses.

**Method:**

A total of 92 nurses newly recruited in 2019 were included in the present study and randomly divided into two groups: the intervention group and the control group (*n* = 46, each). An ordinary training program was applied in the control group, and the flipped classroom + mind map training method was applied in the intervention group. All the new nurses were evaluated using the autonomous learning ability scale before and after pre-job training.

**Results:**

The results of the present study showed that before the pre-job training, the total scores of independent learning ability, learning motivation, self-management ability, learning cooperation ability and information quality of nursing staff were similar in the control group and the intervention group; the differences were not statistically significant (*P* > 0.05). After the application of different training methods, the total score of independent learning ability (84.95 ± 5.146 vs. 66.73 ± 11.213), learning motivation (28.65 ± 3.198 vs. 22.78 ± 5.995), self-management ability (24.97 ± 3.586 vs. 17.89 ± 4.153), learning and cooperation ability (14.391 ± 1.584 vs. 12.17 ± 2.584) and information quality score (16.93 ± 1.306 vs. 13.89 ± 2.651) in the intervention group were significantly higher than in the control group; the differences were statistically significant (*P* < 0.05).

**Conclusion:**

The flipped classroom + mind map training method can effectively improve the autonomous learning ability of newly recruited nurses.

## Introduction

The purpose of pre-job standardised training is enabling new nurses to quickly improve their skills and adapt to clinical work as soon as possible; the training will have an important impact on their future nursing quality and career-planning abilities [1]. Traditional pre-job training mainly provides theoretical lectures and operational skill training for new nurses, this mode mainly focuses on pre-class preparation, independent study, discussion and classroom teaching, supplemented by multimedia and other traditional teaching methods. It has a single teaching method, lacks in comprehensiveness and flexibility, and has unsatisfactory teaching effects [2, 3]. The flipped classroom is a new teaching model in which the traditional teaching process is “flipped”. In this model, students first learn alone before class, then internalise their knowledge through student interaction and teacher–student interaction in class. This changes teachers from knowledge imparters in the traditional classroom to promoters and instructors of learning [4–6]. In recent years, flipped classrooms have achieved good success in multidisciplinary practice and application; this method was found to be more effective in adult learning than traditional didactic lectures [7]. It was further found that it is more enjoyable and provides a more positive motivation in clinical teaching [8, 9].

The mind map, which was originally proposed by British scholar Tony Bazin, is a tool that concretises divergent thinking. It uses vocabulary, lines, symbols and images to form divergent and nodal structural forms. It further transforms tedious text information into hierarchical maps to improve learners’ information storage and extraction as well as their work and learning efficiency [10–13]. The tool is based on radioactive thinking and applies the technique of graphical and textual representation using relevant hierarchical diagrams to show the relationships of topics at all levels. It also makes full use of colour and font changes to visualise a long list of mundane information and results. It can fully mobilise the logic of the left brain as well as the imagination and holistic thinking of the right brain, allowing for the fullest possible brain development [14]. The mind map can be used as a teaching tool to encourage learners to integrate information across disciplines, understand the relationship between basic science and clinical science, and advance medical education [15, 16].

In order to improve and enhance the effectiveness of pre-job training for new nurses, the nursing department of our hospital has been applying the flipped classroom + mind map method for pre-job training since 2019 with good results. Therefore, the present study was conducted with the new 2019 nursing staff in our hospital as the main observation subjects. The present study intends to investigate the effect of the flipped classroom and mind map in the pre-job training of newly nurses.

## Data and methods

### Study subjects

A total of 92 members of nursing staff newly recruited in 2019 in our hospital were selected as the study subjects. Sequences were generated using a random assignment software and stored in sealed, opaque envelopes by an independent worker. Based on the results of a random lottery, the participants were then assigned to two groups: the treatment group and the control group (*n* = 46, each). The treatment group comprised: 12 males and 34 females; 16 junior college graduates and 30 undergraduates; 12 only children and 34 non-only children; 24 class leaders and 22 class non-leaders; and 35 rural residents and 11 urban residents. The mean participant age was 22.58 ± 1.00 years. The control group comprised: 8 males and 38 females; 13 junior college graduates and 33 undergraduates; 8 only children and 38 non-only children; 21 class leaders and 25 class non-leaders; and 32 rural residents and 14 urban residents. The mean participant age was 22.43 ± 0.93 years.

The conventional pre-job training with theoretical knowledge lectures was applied in the control group, and the flipped classroom + mind map training model was applied in the treatment group. The study complied with the *World Medical Association Declaration of Helsinki* and was approved by the ethics committee of our hospital. All participants signed an informed consent form.

### Inclusion and exclusion criteria

#### Inclusion criteria

Inclusion criteria for the present study: (1) Subjects who were newly recruited as nursing staff in 2019; (2) subjects with normal text reading and writing functions who could communicate verbally; (3) subjects with a registered nurse practice licence; (4) subjects aged > 18 years; and (5) subjects who signed the informed consent form.

#### Exclusion criteria

Exclusion criteria for the present study: (1) Subjects who were missed for various reasons; and (2) subjects without a registered nurse practice licence.

### Study methods

This study was conducted in the training room of our hospital.

#### Control group

Conventional pre-job training with theoretical knowledge lectures was applied. I.e. students in the pre-class stage to preview, independent study, discussion, classroom teaching method supplemented by multimedia and other traditional teaching methods for teaching, attendance assessment after class.

#### Treatment group

All training teachers involved in this study received rigorous training. The class duration was set to 1.5–2 h. In the treatment group, the 46 newly recruited nurses were first trained in the mind map application method to understand its concept, background, meaning, and drawing method. Next, they were guided through the design of mind map lesson plans with examples. Then, *Basic Nursing* was used as a reference book for the content of theoretical knowledge involved in the training program, and a task was set aside for the nurses to make a mind map around the content of a chapter or section. Finally, 10 excellent works were selected among 46 works for the authors to show and explain, and the new nurses who were shortlisted to show their works were given extra points for performance. A question-and-answer interaction was set up during each training session: after the content of each guide is explained, the training teacher asks questions about the content of the guide and the extended content involved, and the new nurses raise their hands to answer the questions. Those who answer correctly are given extra points in the classroom; these are recorded by an independent professional.

The score statistics were included in the evaluation of excellent students in pre-job training. Before and after the pre-job training, the self-learning ability of new nurses was evaluated using the *Self-learning Ability Evaluation Scale of Nursing Students*.

After the pre-job training, the clinical assessment of skills, including vital sign monitoring, oxygen inhalation, intravenous infusion, turning over and tapping back auscultation, was organised.

### Research tools

Sociodemographic surveys were conducted to obtain gender, race, age, only child status and class leadership status.

The *Self-learning Ability Evaluation Scale of Nursing Students*, which was developed by Zhang Xiyan in 2007, was widely used to assess the independent learning ability of nursing students. Independent learning ability is a proactive form of learning, in which individuals decide and regulate their own learning behaviors and process according to their own standards without being controlled by others [17]. The scale contains four dimensions: learning motivation, self-management ability, learning cooperation ability, and information quality. It contains 10, 9, 5 and 6 questions, respectively, for a total of 30 items. The overall Cronbach’s α was 0.90, which showed that the scale has good reliability and validity. The self-statement scale was used to assess the self-learning ability of nursing students, and all questions were multiple-choice.

The five-level reaction system of the Likert scale (completely consistent, basically consistent, general, basically inconsistent and completely inconsistent) was used. The scale was scored as follows: 5, 4, 3, 2 and 1 for positive statements, and the reverse for negative statements. The overall Cronbach’s α was 0.86, which showed that the scale has good reliability and validity. The core ranges were: 30–150 in total scale scores, 10–50 in learning motivation scores, 9–45 in self-management scores, 5–25 in learning cooperation scores, and 6–30 in information quality scores. A higher score represented a stronger independent learning ability.

### Statistical methods

The SPSS 25.0 software was used to establish the database and analyse the data. The measurement data were expressed as mean ± standard deviation ($\overline{x }$ ± s), and the counting data were expressed as percentage (%). The W test was used for the normality test, the F test was used to test the homogeneity of variance, the T-test was used for comparison between the two groups with normal distribution, the nonparametric test was used for comparison between the two groups with non-normal distribution, and the Chi-square test was used for counting data. A *P* value of < 0.05 was considered statistically significant.

## Results

### General data

The statistical results showed that there was no statistical difference between the two groups in terms of age, gender, education, family residence, whether they had served as class leaders, and whether they were only children (*P* < 0.05). Therefore, the two groups were comparable. The specific results are shown in Table 1.

**Table 1:** Comparison of general demographic data between the two groups of new nursing staff

project	category	Intervention group		control group		X^2^/t Value	*P*
		*n* = 46	%	*n* = 46	%		
Age		22.58 ± 1.00		22.43 ± 0.93		0.753	0.453
Gender	male	12	26.1	8	17.4	1.022	0.312
	female	34	73.9	38	82.6		
education background	Specialized subject	16	38.4	13	28.3	0.453	0.501
	Undergraduate course	30	61.6	33	71.7		
Only child or not	Yes	12	26.1	8	17.4	1.022	0.312
	No	34	73.9	38	82.6		
Whether appointment class cadre	Yes	24	52.2	21	45.7	0.391	0.532
	No	22	47.8	25	54.3		
Family residence	rural	35	76.1	32	69.6	0.495	0.482
	city	11	23.9	14	30.4		

### Comparison of independent learning ability before and after pre-job training in the control group

In the control group, the mean score of total independent learning ability was 63.06 ± 11.524 before pre-job training and 66.73 ± 11.213 after pre-job training; the difference was not statistically significant (*P* > 0.05). In addition, there was no statistically significant difference in the scores of learning motivation, self-management ability, learning cooperation ability and information quality in the control group before and after the pre-job training (*P* > 0.05). The results are shown in Table 2.

**Table 2:** Comparison of scores of independent learning ability before and after pre-service training for newly recruited nurses in the control group

Project	Autonomous Learning Ability	Learning motivation	Self management ability	Learning cooperation ability	Information literacy
Before pre job training	63.06 ± 11.524	20.60 ± 6.075	17.26 ± 4.533	11.65 ± 2.892	13.54 ± 2.986
After pre job training	66.73 ± 11.213	22.78 ± 5.995	17.89 ± 4.153	12.17 ± 2.584	13.89 ± 2.651
t	-1.550	-1.727	-0.695	-0.912	-0.591
p	0.125	0.088	0.489	0.364	0.556

### Comparison of independent learning ability before and after pre-service training in the treatment group

In the treatment group, the mean score of total independent learning ability was 65.28 ± 10.540 before pre-job training and 84.95 ± 5.146 after pre-job training, the difference was statistically significant (*P* < 0.05). In addition, there was a statistically significant difference in scores of learning motivation, self-management ability, learning cooperation ability and information quality before and after the pre-job training (*P* < 0.05). The results are shown in Table 3.

**Table 3:** Comparison of scores of independent learning ability before and after pre-job training for newly recruited nurses in the pre-job training group

Project	Autonomous Learning Ability	Learning motivation	Self management ability	Learning cooperation ability	Information literacy
Before pre job training	65.28 ± 10.540	20.04 ± 4.075	18.39 ± 3.985	12.01 ± 2.296	14.13 ± 2.334
After pre job training	84.95 ± 5.146	28.65 ± 3.198	24.97 ± 3.586	14.391 ± 1.584	16.93 ± 1.306
t	-11.376	-10.459	-8.331	-4.069	-7.110
p	0.000	0.000	0.000	0.000	0.000

### Comparison of the independent learning ability of nursing staff in the two groups after pre-job training

The statistical results showed that the scores of all independent learning ability dimensions after pre-job training were significantly higher in the treatment group than in the control group; the differences were statistically significant (*P* < 0.05). The specific results are shown in Table 4. Two groups of nursing staff clinical skills assessment all qualified.

**Table 4:** Comparison of scores of self-learning ability between the two groups after pre job training

Project	Autonomous Learning Ability	Learning motivation	Self management ability	Learning cooperation ability	Information literacy
Before pre job training	66.73 ± 11.213	22.78 ± 5.995	17.89 ± 4.153	12.17 ± 2.584	13.89 ± 2.651
After pre job training	84.95 ± 5.146	28.65 ± 3.198	24.97 ± 3.586	14.391 ± 1.584	16.93 ± 1.306
t	-10.014	-5.858	-8.758	-4.961	-6.983
p	0.000	0.000	0.000	0.000	0.000

## Discussion

A total of 92 new members of nursing staff were included in this study and randomly divided into the control group and the treatment group. Conventional pre-job training with theoretical knowledge lectures was applied in the control group, while the flipped classroom + mind map training model was applied in the treatment group. The results of this study showed that before the pre-job training, the total score of independent learning ability and the scores of learning motivation, self-management ability, learning cooperation ability and information quality were similar in the control group and the treatment group; the differences were not statistically significant. However, after adopting different training methods, the total scores of independent learning ability, learning motivation, self-management ability, learning cooperation ability and information quality were significantly higher in the treatment group than in the control group; the differences were statistically significant.

This study shows that applying the flipped classroom and mind map in pre-job training can effectively improve the independent learning ability of new nurses. New nurses will read through and integrate the textbook knowledge to draw their mind maps; this will invariably improve their independent learning abilities. By giving bonus points for answering questions in the classroom questioning link and incorporating them into the performance appraisal of excellent pre-job trainees’ evaluation results, the motivation of new nurses’ learning is stimulated. This has opened up ideas for improving nurses’ autonomous learning abilities in the entire hospital.

In pre-job training using the flipped classroom method, the use of mind maps is beneficial to the optimisation of teaching content [15–17]. Through mind map presentation, a more concise and clear language context can be created, effective hints and associations of keywords can be made, and the relevance of knowledge can be constructed; this is conducive to the expansion of new nurses’ thinking and helps them clarify their thoughts, thus integrating them more actively into the flipped classroom model [18–20]. Compared with the general teaching format, new nurses can carry out dialogue communication more fluently and with richer dialogue communication after mind map application. Hence, new nurses are motivated to participate in the flipped classroom model, and a good foundation is laid down for the deepening of the training content.

The application of the flipped classroom + mind map training model in pre-job training captured the attention of new nurses in the classroom [21–23], and through the teacher’s questions and answers in the classroom and addition to the classroom grade scoring statistics, the students’ enthusiasm was greatly mobilised [13, 24]. The same phenomenon was noted in the treatment group of this study. There was no more cross-talk or desertion to use cell phones in the classroom. The nurses were able to devote themselves to the training course designed in the pre-job training.

This study still has several shortcomings: (1) the study is a randomised controlled trial. However, it is not blinded, so there is still some risk of bias; (2) the study is a single-centre clinical study, and a multi-centre clinical study is still needed for further exploration in the follow-up; and (3) the sample size is small, and further studies with larger sample sizes are still needed.

## Conclusion

This study further elaborates the practical value of flipped classroom + mind map application in pre-job training to enhance new nurses’ independent learning abilities, optimise training content, and improve classroom interaction. Therefore, in the actual training, teachers need to deeply explore the application value of the flipped classroom + mind map model; closely integrate with pre-job training reform; give full play to the intuitive, guiding, expanding and reinforcing features of the flipped classroom + mind map model; effectively enhance the flexibility and timeliness of training; and explore new ways of reform in the pre-job training of new nurses.

## Data Availability

The datasets used and/or analysed during the current study are available from the corresponding author on reasonable request.

## References

[1] Liu HH (2019). Evaluation of the effect of one-to-one pre-job standardized training for new nurses in hemodialysis room. China Health Ind.

[2] Dobrowolska B, McGonagle I, Kane R (2016). Patterns of clinical mentorship in undergraduate nurse education: a comparative case analysis of eleven EU and non-EU countries. Nurse Educ Today.

[3] Stefaniak M, Dmoch-Gajzlerska E (2020). Mentoring in the clinical training of midwifery students - a focus study of the experiences and opinions of midwifery students at the Medical University of Warsaw participating in a mentoring program. BMC Med Educ.

[4] Schwartz TA (2014). Flipping the Statistics Classroom in Nursing Education. J Nurs Educ.

[5] Hessler KL (2016). Nursing education: Flipping the classroom[J]. Nurse Pract.

[6] Billings DM (2016). 'Flipping' the classroom. Am J Nurs.

[7] Rose E, Claudius I, Tabatabai R (2016). The flipped classroom in emergency medicine using online videos with interpolated questions. J Emerg Med.

[8] Dooley LM, Frankland S, Boller E (2018). Implementing the flipped classroom in a veterinary pre-clinical science course: student engagement, performance, and satisfaction. J Vet Med Educ.

[9] Mortensen CJ, Nicholson AM (2015). The flipped classroom stimulates greater learning and is a modern 21st century approach to teaching today's undergraduates. J Anim Sci.

[10] Ahn HY, Yeon E, Ham E (2006). Patient modeling using mind mapping representation as a part of nursing care plan.

[11] Wu HZ, Wu QT (2020). Impact of mind mapping on the critical thinking ability of clinical nursing students and teaching application. J Int Med Res.

[12] Yang H, Gao X, Li MH (2020). The use of mind mapping in health education in extended care for children with caries. J Int Med Res.

[13] Kotcherlakota S, Zimmerman L, Berger AM (2013). Developing scholarly thinking using mind maps in graduate nursing education. Nurse Educ.

[14] Guerrero JM (2015). Introduction to the Applications of Mind Mapping in Medicine.

[15] D'Antoni AV, Zipp GP, Olson VG (2010). Does the mind map learning strategy facilitate information retrieval and critical thinking in medical students?. BMC Med Educ.

[16] Farrand P, Hussain F, Hennessy E (2002). The efficacy of the 'mind map' study technique. Med Educ.

[17] Papamitsiou Z, Economides AA (2019). Exploring autonomous learning capacity from a self-regulated learning perspective using learning analytics. Br J Educ Technol.

[18] Della Ratta CB (2014). Flipping the classroom with team-based learning in undergraduate nursing education. Nurse Educ.

[19] El-Banna MM, Whitlow M, Mcnelis AM (2017). Flipping around the classroom: accelerated Bachelor of Science in Nursing students' satisfaction and achievement. Nurse Educ Today.

[20] Betihavas V, Bridgman H, Kornhaber R (2016). The evidence for 'flipping out': a systematic review of the flipped classroom in nursing education. Nurse Educ Today.

[21] Spencer JR, Anderson KM, Ellis KK (2013). Radiant thinking and the use of the mind map in nurse practitioner education. J Nurs Educ.

[22] Schlairet MC, Green R, Benton MJ (2014). The flipped classroom: strategies for an undergraduate nursing course. Nurse Educ.

[23] Rooda LA (1994). Effects of mind mapping on student achievement in a nursing research course. Nurse Educ.

[24] Yu L, Zhou B, Xu S (2017). Mind maps for the application of ENT nursing teaching in higher vocational colleges. Today Nurse.

[25] Pence PL, Franzen SR, Kim MJ (2021). Flipping to motivate: perceptions among prelicensure nursing students. Nurse Educ.

[26] Hessler K (2016). Flipping the nursing classroom: where interactive learning meets technology.

